# Atherosclerosis endothelial activation quantification in vivo with fluorine magnetic resonance imaging and spectroscopy

**DOI:** 10.1186/1532-429X-16-S1-O91

**Published:** 2014-01-16

**Authors:** Hua Pan, Jacob Myerson, Xiaoxia Yang, Gregory Lanza, Samuel A Wickline

**Affiliations:** 1 Washington Univeristy School of Medicine, Saint Louis, Missouri, USA

## Background

Endothelial activation is one of the necessary initial steps in the formation of atherosclerotic plaque that facilitates immune cell recruitment and retention. To image and quantify early markers of endothelial inflammation, we recently developed a multiplexing peptide-based targeting system for post-formulation functionalization of perfluorocarbon (19F) nanoparticles (PFC NP) that is intrinsically quantifiable at a binding site based on magnetic resonance spectroscopy. The approach is now demonstrated in vivo to detect and quantify VCAM-1 in ApoE-/- mouse aorta.

## Methods

VCAM-1 targeted PFC NPs were generated through post-formulation strategy by using linker peptides. Specific targeting has been validated in vitro by using confocal imaging, 19F magnetic resonance spectroscopy (MRS), and magnetic resonance imaging (MRI). Quantification of endothelial activation in atherosclerosis has been investigated by using ApoE-/- and control C57-BL6 mice through MRS.

## Results

Specific targeting to VCAM-1 in vitro to mouse endothelial cells subjected to TNF-α stimulation was confirmed by confocal imaging and magnetic resonance imaging (MRI), and quantified by 19F magnetic resonance spectroscopy (MRS) (11.7T) in comparison to a 19F calibration standard. Next, ApoE-/- and control C57-BL6 mice (n ≥3/group) fed a western diet for 8 weeks, were injected i.v. with VCAM-1 targeted or non-targeted nanoparticles. After two hours, aortas were subjected ex vivo to MRS analysis (11.7T) which revealed a 4.6-fold increase in the amount of nanoparticles in aortas from the ApoE-/- mice receiving VCAM-1 targeted nanoparticles as compared with other groups (ApoE -/- mice: targeted vs non-targeted - 8.22 ± 4.4 vs 1.78 ± 1.2 × 105 NPs/mm2, p < 0.05; C57-BL6 mice: targeted vs non-targeted - 2.28 ± 1.24 vs 2.08 ± 0.98 × 107 NPs/mm2).

## Conclusions

The use of 19F MRI/MRS together with a flexible and rapid postformulation approach to creating ligand targeted nanoparticulate contrast agents may enhance the efficiency and breadth of molecular imaging of atherosclerosis with MRI and enable quantification of selected biomarker molecules that are important early drivers of atherosclerosis.

## Funding

HL-073646 and CA-119342 to S.A.W.

